# Spectroscopic Characterization of i-motif Forming c-myc Derived Sequences Double-Labeled with Pyrene

**DOI:** 10.1007/s10895-013-1184-z

**Published:** 2013-03-22

**Authors:** Anna Dembska, Patrycja Rzepecka, Bernard Juskowiak

**Affiliations:** Laboratory of Bioanalytical Chemistry, Faculty of Chemistry, A. Mickiewicz University, Umultowska 89b, 61-614 Poznań, Poland

**Keywords:** i-motif, Fluorescence, pH, Oligonucleotide probe, Pyrene

## Abstract

In current studies we use the oligonucleotides based on c-myc sequence: CCC CAC CCT CCC CAC CCT CCC C (cmyc22) and CCC CAC CCT CCC CAC CCT CCC CA (cmyc22A) functionalized by pyrene moieties at both termini. Results of the circular dichroism (CD), UV absorption melting experiments, and steady-state fluorescence measurements of pyrene-modified i-motifs as well as their unlabeled precursors are presented and discussed here. The pyrene labels have a remarkable influence on i-motif stability which was deduced from CD spectra and confirmed by UV melting experiments. Both probes emit fluorescence band of pyrene monomer with intensity decreasing upon pH lowering.

**Electronic supplementary material:**

The online version of this article (doi:10.1007/s10895-013-1184-z) contains supplementary material, which is available to authorized users.

## Introduction

A lot of important genomic regions, especially in gene promoters consist of repeating sequences potentially able to form tetraplexes on both DNA strands [1]. Precisely speaking, G-rich strands are known to form G-quadruplexes, whereas complementary C-rich strands can adopt i-motif structure [2–6]. Folding into i-motif architecture is much more complicated/demanding process since it must be preceded by the protonation of cytosines [7–10]. However, the latest investigations proved that i-motifs are present not only at slightly acidic but even in neutral and slightly alkaline pH [11, 12]. The feature of sequences that include tracks of cytosines to switch from folded i-motif to random coil in response to pH changes were used, by a few research groups, as an important part of the interesting biosensors also called nanomachines [13–17]. The conformational sensitivity of C-rich oligonucleotides to pH changes encourages also us to design and investigate ability of intramolecular i-motif based probes for pH monitoring. In our approach, the intramolecular i–tetraplex building oligonucleotide is modified by pyrene tags at the both ends. Pyrene and its derivatives are known of ability to exhibit excimer fluorescence induced by stacking interactions between at least two aromatic rings [18]. This phenomenon was explored to create a wide range of sensors [19–22] including one, called PSO-py, based on G-quadruplex forming thrombin-binding aptamer [23]. PSO-py probe for K^+^ monitoring is just a dual-pyrene-labeled G-rich oligonucleotide that undergoes folding into G-quadruplex in the presence of K^+^. The three-dimensional (3D) architecture of PSO-py/K^+^ complex brings on stacking interactions between fluorophores (pyrene) and produce excimer emission. The simplicity of this light switch system is its indisputable advantage. Seeing some similarities between topology of the PSO-py/K + chair-type quadruplex and the intramolecular i-motif, we are convinced that our idea of creating the analogues sensors for pH monitoring based on intramolecular i-motifs is worth explorations.

**Fig. 1:**
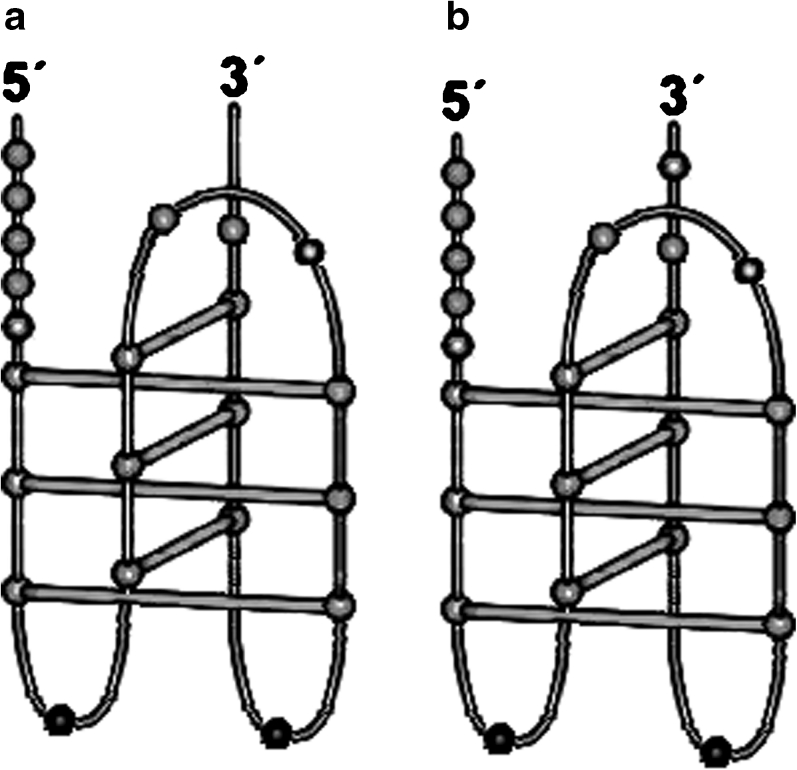
Structure of intramolecular i-motifs formed by: **a** - CCC CAC CCT CCC CAC CCT CCC C (cmyc22) and **b** - CCC CAC CCT CCC CAC CCT CCC CA (cmyc22A)

The presented paper is a part of our project focus to examine the capability of different i-motifs to serve as a pH sensor. As mentioned above, it is important to select a sequence which was proved to form only intramolecular fold-back i-motifs. The well characterized sequence known to form intramolecular i-tetraplex is 22-mer from the human c-myc gene corresponding to bases 2190–2211 of the locus [24]. Simonsson et al. [24] proposed the structure of i-motif formed by this sequence, which is presented in Fig. 1a. The next natural base (number 2212) in cmyc gene is adenine as it is shown in Fig. 1b. We decided to examine both these sequences as an oligonucleotide part of the designed probe to check the effect of additional nucleobase on the probe characteristics.

**Fig. 2:**
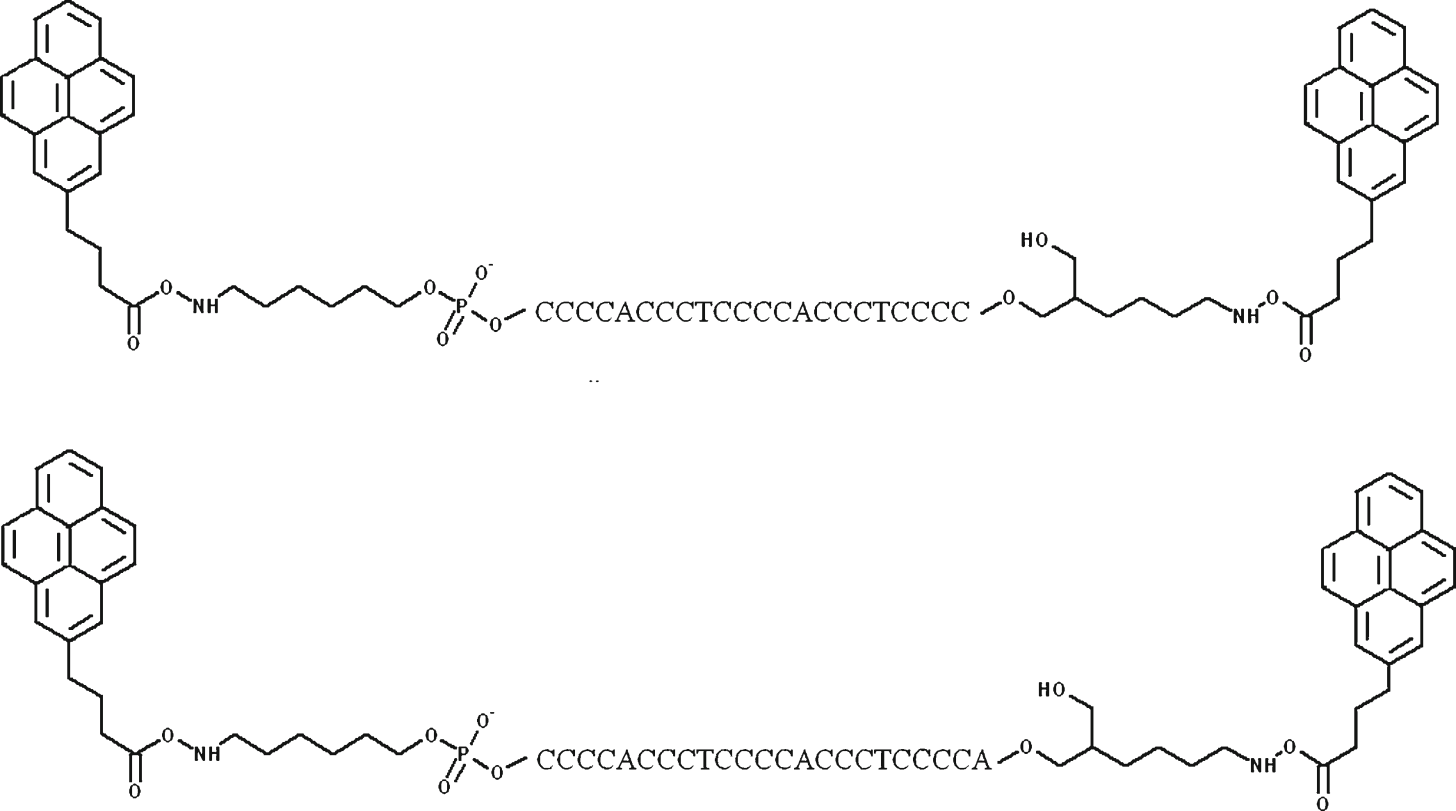
The illustration of pyrene attachment to cmyc22 (*upper*) and cmyc22A (*lower*) sequences

We linked pyrene to both ends of chosen sequences: 5′-CCC CAC CCT CCC CAC CCT CCC C-3′ and 5′-CCC CAC CCT CCC CAC CCT CCC CA-3′ obtaining fluorescent probes called Py-cmyc22-Py and Py-cmyc22A-Py, respectively (Fig. 2). The attachment of pyrene moieties to oligonucleotides was done according to one-step post-synthetic procedure, as reported by Kierzek at al. [25]. Results of the circular dichroism (CD), UV melting experiments and steady-state fluorescence measurements these of pyrene-modified i-motifs are presented and discussed here.

## Experimental

### Materials

Unlabeled oligonucleotides were custom synthesized by Genomed SA (Poland). The oligonucleotides: CCC CAC CCT CCC CAC CCT CCC C and CCC CAC CCT CCC CAC CCT CCC CA with modification called 5′ C6-Aminolink and 3′ C7- Aminolink were purchased from Generi Biotech (Czech Republic). All oligonucleotides were HPLC-purified. Py-cmyc22-Py and Py-cmy22A-Py were synthesized according to the procedure described by Kierzek et al. [25] and were purified by means of HPLC. Other reagents were of analytical grade purity and were used as received. The buffer used in the work was 10 mM Tris adjusted to desired pH by acetic acid unless otherwise stated. High-purity water (Polwater, Poland) was used throughout. The strands concentration were determined by UV absorbance at 260 nm at a high temperature (above 85 °C), assuming the molar extinction coefficients of 7400 M^−1^ cm^−1^for cytosine, 15400 M^−1^ cm^−1^ for adenine and 8700 M^−1^ cm^−1^ for thymine. Before spectral measurements, the solution of 1 μM DNA (in strand units) in appropriate buffer solutions were annealed by being heated to 90 °C and then slowly cooled to room temperature.

### CD Spectroscopy

CD spectra were recorded on a Jasco J-810 spectropolarimeter at room temperature. Each measurement was the average of four repeated scans recorded from 230 to 340 nm with a 10 mm quartz cell at a scan speed of 200 nm/min. The scan of the corresponding buffer solution was subtracted from the average scan for each sample.

### UV Melting Measurements

UV melting profiles were recorded on a Cary 100 Biomelt (Agilent Technologies) spectrophotometer equipped with a Peltier temperature control accessory. The all measurements were done in a quartz cell with a path length of 1.0 cm. UV melting curves were measured by monitoring the absorbance at 260 nm, while the temperature was increased at a rate of 1 °C/min. The melting temperature of i-motif was obtained from the absorbance at 260 nm plotted *versus* temperature. The obtained curves were analyzed using nonlinear regression to evaluate the melting temperatures.

### Fluorescence Spectroscopy

Steady-state fluorescence measurements were carried out on a spectrofluorometer model JASCO FP – 6200 at room temperature. Spectra were collected from 370 to 620 nm while exciting at 340 nm. In these measurements, the path lengths of the quartz cell used were 0.2 cm in the excitation direction and 1 cm in the emission direction. All emission spectra were uncorrected.

## Results

Syntheses of Py-cmyc22-Py and Py-cmyc22A-Py probes (Fig. 2) were carried out according to Kierzek et al. [23]. Products were purified by HPLC. We first determined how pyrene moieties attached to both ends of Py-cmyc22-Py and Py-cmyc22A-Py influence the formation and stability of i-motifs. CD and UV spectroscopy measurements were performed at various pH values and compared with results obtained for unlabeled precursors.

**Fig. 3:**
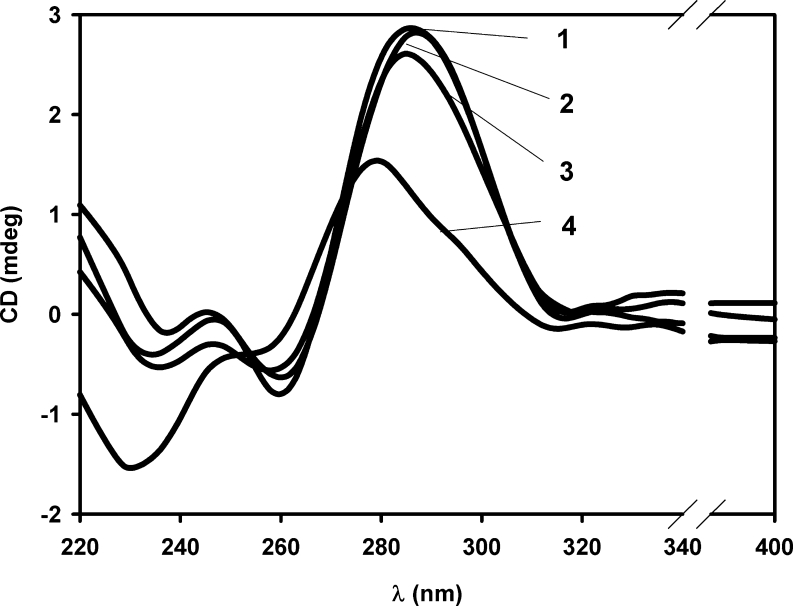
CD spectra of Py-cmyc22-Py at the different pH values: pH 5.5 (line 1), pH 6.5 (line 2), pH 7.0 (line 3) and pH 7.5 (line 4)

**Fig. 4:**
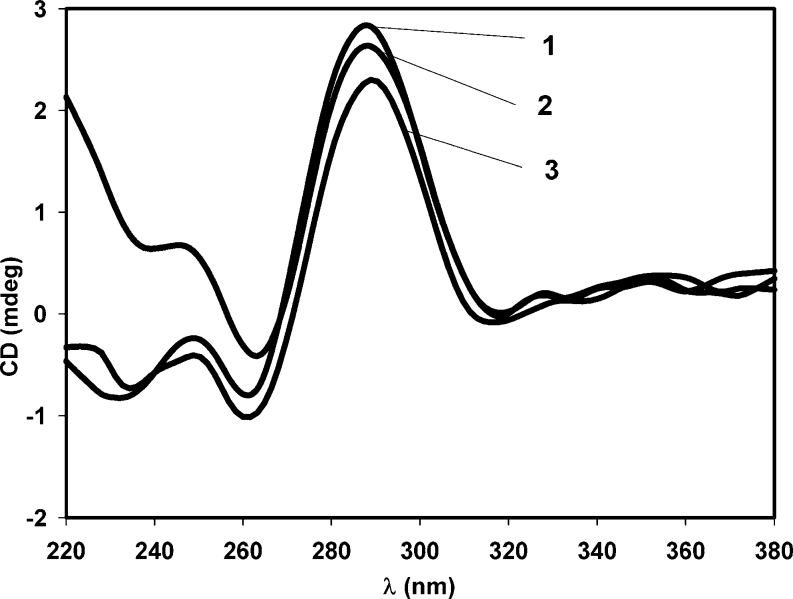
CD spectra of Py-cmyc22A-Py at the different pH values: pH 5.5 (line 1), pH 6.5 (line 2), pH 7.0 (line 3)

The CD spectra of Py-cmyc22-Py and Py-cmyc22A-Py in Tris-acetate buffers at pH range 4.0–7.0 are almost identical, therefore; only representative results at pH 5.5, 6.5 and 7.0 are illustrated in Fig. 3 and Fig. 4. CD spectra consist of one very sharp positive band at 288 nm and weak negative band at 262 nm. These two bands are characteristic for an i-motif secondary structure [4, 8, 24]. However, it is unusual that negative peak is so weak and about 4 times less intensive than positive one. In case of unlabeled sequence, we observed very sharp negative and positive peaks, which started to loose intensities when pH increased and finally at pH around 7.0 the positive maximum shifted toward 277 nm indicating unfolding i-motif structure (Fig. S1 and Fig. S2). Surprisingly, secondary structures of Py-cmyc22-Py and Py-cmyc22A-Py are much more stable - there are almost no changes in the position and intensity of the CD minima and maxima up to pH 7.0 for Py-cmyc22-Py and to pH 8.0 for Py-cmyc22A-Py (data not shown). For the latter case we were not able to indicate pH-transition midpoint. The pH of structural transition for Py-cmyc22-Py is located between 7.0 and 8.0 because the positive maximum begins to shift toward 277 nm at pH 7.5 (Fig. 3).

**Fig. 5:**
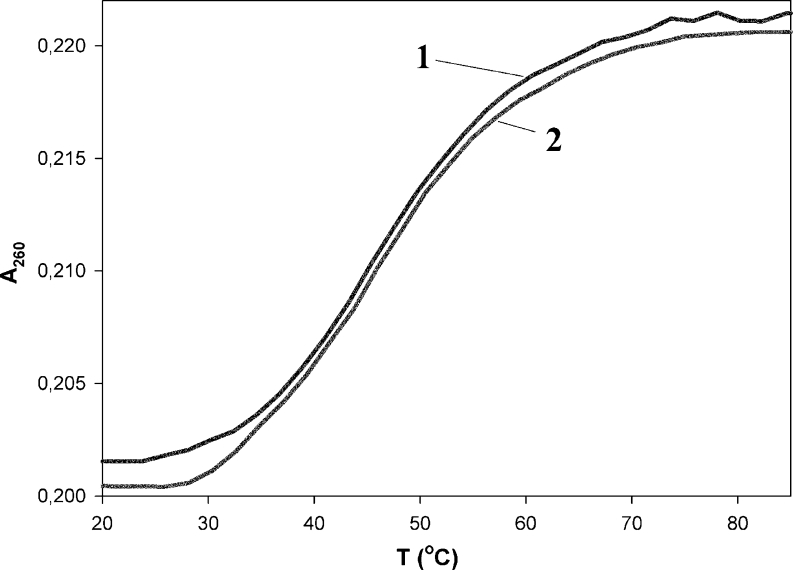
The melting profile of Py-cmyc22-Py at the pH 6.5. The presented curves are cooling (line 1) and heating (line 2)

**Fig. 6:**
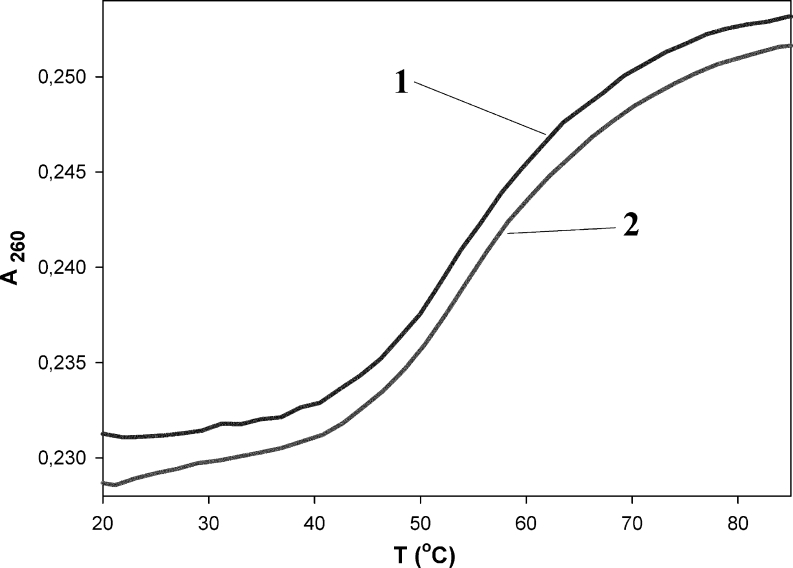
The melting profile of Py-cmyc22A-Py at the pH 6.5. The presented curves are cooling (line 1) and heating (line 2)

UV melting curve experiments were also performed in order to investigate the thermal stability of Py-cmyc22-Py and Py-cmyc22A-Py in different pH values. The examples of thermal profiles are presented in Figs. 5 and 6, which were generated by plotting mole fraction of folded probes versus temperature. The renaturating and denaturating processes were reversible at the heating and cooling rate of the experiments (1 °C per 1 min). A transition between two states (folded and unfolded) seems to be highly cooperative, as expected for intramolecular i-motif [24, 26]. The evaluated Tm values are collected in Table 1. For labeled as well as unlabeled sequences, the maximum melting temperatures were obtained at pH 4.5, which is close to the pKa of cytosine (4.8) [26]. Py-cmyc22-Py melts in very similar way to its unlabeled precursor – Tm in pH range 4.0-6.5 are within 2 °C, whereas by 7 °C degrees higher at pH 7.0. Py-cmyc22A-Py melts at lower temperature at pH below 5.5 than identical sequence without pyrene moieties at the both ends. However, we observed higher melting temperatures for Py-cmyc22A-Py at pH above 5.5 and sigmaidol shape of melting profiles was observed even at pH 8.0 (Fig. S3).

**Table 1:** The melting temperatures (Tm) evaluated from denaturating profiles obtained at different pH values

*Oligonucleotide*		Tm (°C)								
	pH	4.0	4.5	5.0	5.5	6.0	6.5	7.0	7.5	8.0
*cmyc22*		60.4	60.5	60.0	50.3	50.8	43.0	31.9	n/d	n/d
*Py-cmyc22-Py*		62.5	62.6	61.4	52.2	50.1	42.8	39.0	29.1	n/d
*cmyc22A*		58.5	63.6	61.7	56.4	45.5	41.2	24.5	n/d	n/d
*Py-cmyc22A-Py*		57.8	57.7	55.2	53.6	55.8	52.1	50.3	49.2	34.0

*N/d* not detectable

**Fig. 7:**
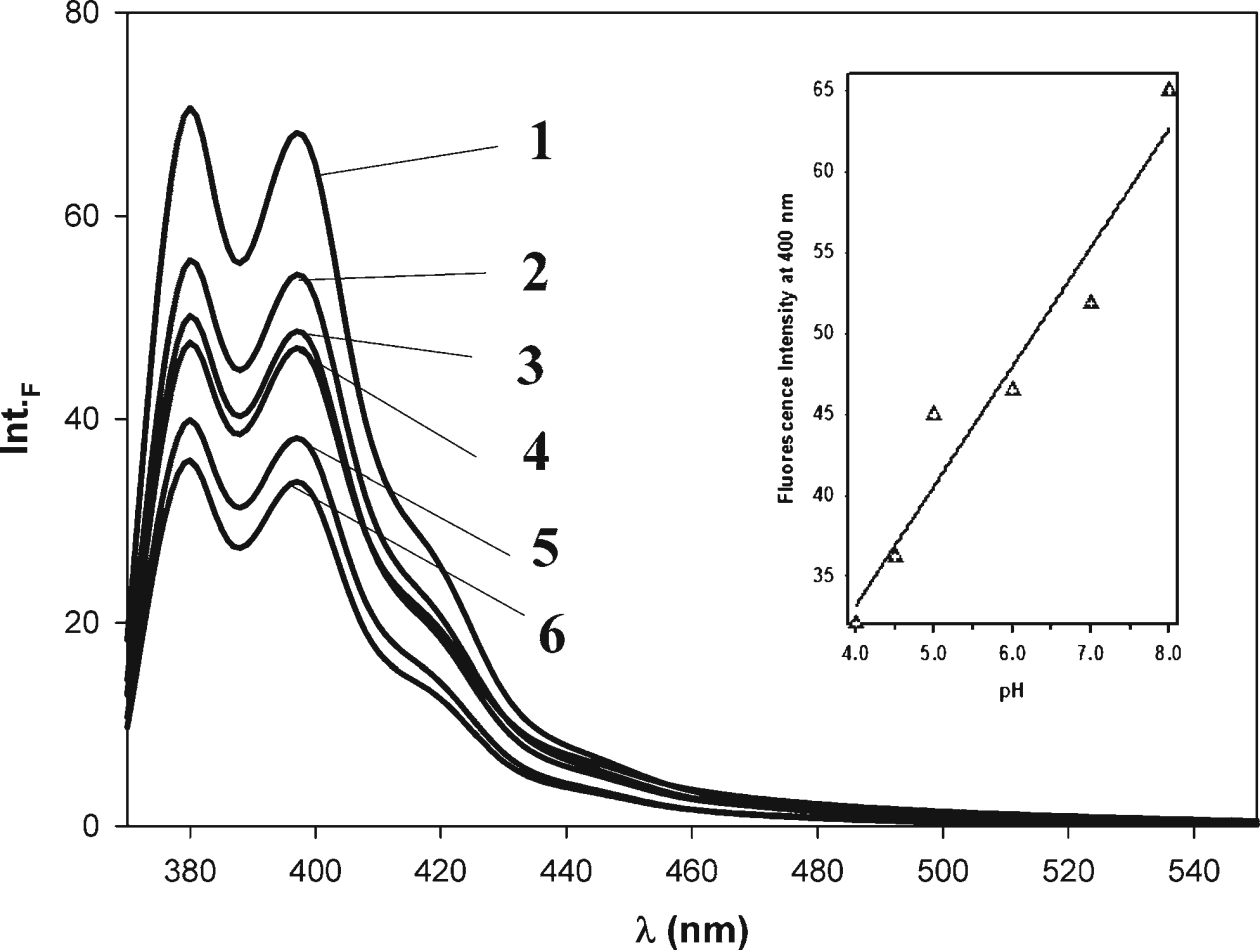
The emission spectra of Py-cmyc22-Py measured at room temperature; at the pH range from 4.0 to 8.0: pH 8.0 (line 1), pH 7.0 (line 2), pH 6.0 (line 3), pH 5.0 (line 4), pH 4.5 (line 5), pH 4.0 (line 6). Insert: The dependence of fluorescence intensity at 400 nm on pH values

**Fig. 8:**
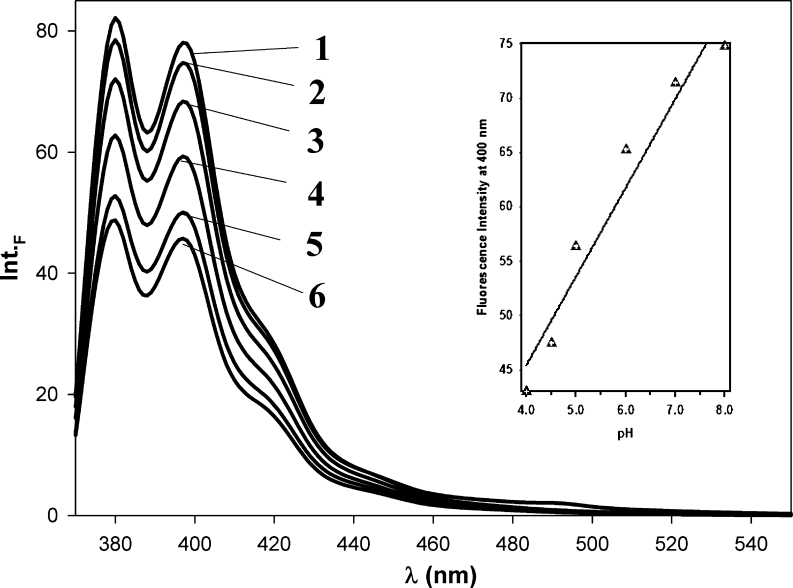
The emission spectra of Py-cmyc22A-Py measured at room temperature; the pH range from 4.0 to 8.0: pH 8.0 (line 1), pH 7.0 (line 2), pH 6.0 (line 3), pH 5.0 (line 4), pH 4.5 (line 5), pH 4.0 (line 6). Insert: The dependence of fluorescence intensity at 400 nm on pH values

We measured steady-state fluorescent spectra of Py-cmyc22-Py probe and Py-cmyc22A-Py in solutions from pH 4.0 to 8.0 (Figs. 7 and 8). We observed a typical emission characteristic for pyrene monomer with a little broadened vibrational shoulder at 420 nm. Monomer fluorescence band at around 380 nm decreased almost by 50 % upon pH lowering from 8.0 to 4.0. The quenching of pyrene monomer emission was not accompanied by raising excimer emission.

## Discussion

It is evident from the data presented above that attachment of two pyrene moieties to C-rich sequences does not affect folding ability of the modified oligonucleotides to form i-motif. The shape of CD spectra of Py-cmyc22-Py and Py-cmyc22A-Py are the same as reported for typical i-motif structure. We think that lower intensity of negative band is caused by pyrene rings attached to both ends of oligonucleotides. On the other hand, the pyrene labels have a remarkable influence on i-motif stability which was deduced from CD spectra and confirmed by UV-melting experiments. What interesting, from changes in ellipicity of the positive band at 288 nm for Py-cmyc22A-Py we were unable to evaluate pH value corresponding to a transition mid-point. Simply, there was lack of changes in CD spectra up to pH 8.0. These results are in good agreement with thermal profiles obtained: for Py-cmyc22A-Py, which have sigmoidal shape in whole range of pH from 4.0 to 8.0. The i-motif structure of Py-cmyc22A-Py is stable even in alkaline solution at room temperature as it melting temperature is 34.0 °C at pH 8.0 and by 10 °C higher at physiological pH (Table 1). High stability of Py-cmyc22A-Py cannot be caused by additional base at 3’ end. In our previous work we indicated that one additional base (thymine or adenine) at 3′ terminus of 5′-CCC CAC CCT CCC CAC CCT CCC C CCC-3′ have no influence on melting temperature [27]. On the other hand, it seems that presence of pyrene at the both termini of C-rich sequence is not enough to induce the greater stability of formed i-motif. The Py-cmyc22-Py melts almost at the same temperature as its unlabeled precursor; except for pH 7.0, at which cmyc22 melts at 7 °C degree lower temperature (Table 1). The most important fact is that although at pH above 7.5 we observe hyperchromicity of absorption band upon increasing temperature, there was no cooperativity in this process in contrast to Py-cmyc22A-Py (Fig. S3 vs. Fig. S4). We can only speculate that additional adenine at 3’ end is able to form hydrogen bond with cytosine present in the loop and this interaction is somehow favored by pyrene rings linked to the ends of oligomer. It is worth to mention that Simonsson et al. [24] proposed architecture of cmyc22 which forms intramolecular i-motif with a long loop consists of TCCCA.

Such long loop present in i-motif can be reason of observed quenching of pyrene monomer fluorescence and a lack of excimer fluorescence around 480 nm. Pyrene is known to exhibit long-wavelength fluorescence attributed to excimer formation, which is possible only in a face-to-face spatial orientation of pyrene rings [18]. When designing the probe, we assumed that the i-motif architecture would be suitable to help pyrene tags, attached to both ends of C-rich oligomer, to form such a sandwich structure. As consequence, i-motif complex should emit excimer fluorescence, as it was observed in case of dual-pyrene-labeled G-rich oligonucleotide folded into G-quadruplex in the presence of K^+^ ion [23]. One of our concern was that the difference in the length of free ends hanging after i-motif formation (Fig. 1) could stand in the way of pyrene residues to meet in desire orientation. To prevent that problem we added adenine or thymine base [27] at the 3’end of cmyc22 sequence. However, this approach was unsuccessful and we did not observe strong excimer fluorescence with maximum around 480 nm. As we said before, it could be caused by the long loop, which could separate pyrene tags or quenched pyrene excited state by steric interaction. This hypothesis should be proved by molecular modeling or by spectroscopic characterization (including the detailed fluorescence lifetime study) of set of dual-pyrene labeled i-motifs based on cmyc22 sequence possessing different spacers of adenines or thymines attached to 3′ and 5′ termini.

Py-cmyc-Py and Pycmyc22A-Py emit only monomer fluorescence with intensities decreasing upon pH lowering. These results support the idea that C-rich sequences functionalized by pyrene at both ends give analytical response upon pH changes.

## Conclusions

Although we observed only pH-sensitive monomer emission of pyrene covalently attached to i-motif forming sequence, we are convinced that intramolecular i-motifs are good foundation for designing simple pH-sensitive fluorescent probes. Such kind of probes are more economical alternative to complicated DNA nano-machines and their analytical response should be better/faster as it is not dependent on many events happening one after another.

On the other hand, the developing and understanding of the exact architecture of i-motif structures at various pH values in the absence of complementary strands is considered as an important challenge in the design of DNA-nanoactuator machines and biological cycle operating systems [28]. The dual-pyrene-functionalized i-motifs give possibility to detail studies on the folding kinetics and dynamics of the conformational changes by using such techniques as time-resolved emission fluorescence, nanosecond time-resolved flash photolysis and transient absorption measurements.

## Electronic supplementary material

Below is the link to the electronic supplementary material.

ESM 1 (DOC 416 kb)
