# Supplementary material for: Spectroscopic Characterization of i-motif Forming c-myc Derived Sequences Double-Labeled with Pyrene
Source: J Fluoresc. 2013 Mar 22;23(4):807–12. doi: 10.1007/s10895-013-1184-z (PMC3696180; doi:10.1007/s10895-013-1184-z)
Supplement: Supplementary file 1 — (DOC 416 kb) [file 10895_2013_1184_MOESM1_ESM.doc]

# Supplementary data for

Spectroscopic characterization of i-motif forming c-myc derived sequences double-labeled with pyrene.

Anna Dembska, Patrycja Rzepecka, Bernard Juskowiak

Laboratory of Bioanalytical Chemistry, Faculty of Chemistry, A. Mickiewicz University, Umultowska 89b, 61-614 Poznań, Poland

Corresponding author: Anna Dembska; e-mail: [*aniojka@amu.edu.pl*](mailto:aniojka@amu.edu.pl)

**Fig. S1** CD spectra of unlabeled cmyc22 at the different pH values: pH 5.5 (line 1), pH 6.5 (line 2), pH 7.0 (line 3) and pH 8.0 (line 4)

**Fig. S2** CD spectra of unlabeled cmyc22A at the different pH values: pH 5.5 (line 1), pH 6.5 (line 2), pH 7.0 (line 3) and pH 8.0 (line 4)

**Fig. S3** The melting profile of Py-cmyc22A-Py at pH 8.0. The presented curves are cooling (line 1) and heating (line 2)

**Fig. S4** The melting profile of Py-cmyc22-Py at pH 8.0. The presented curves are cooling (line 1) and heating (line 2)
